# Comparison of sulfolane effects in Sprague Dawley rats, B6C3F1/N mice, and Hartley guinea pigs after 28 days of exposure via oral gavage

**DOI:** 10.1016/j.toxrep.2021.02.004

**Published:** 2021-02-06

**Authors:** K.A. Shipkowski, M.C. Cora, M.F. Cesta, V.G. Robinson, S. Waidyanatha, K.L. Witt, M.K. Vallant, D.M. Fallacara, M.R. Hejtmancik, S.A. Masten, S.D. Cooper, R.A. Fernando, C.R. Blystone

**Affiliations:** a Division of the National Toxicology Program, National Institute of Environmental Health Sciences, Research Triangle Park, NC, USA; b Battelle Memorial Institute, Columbus, OH, USA; c RTI International, Research Triangle Park, NC, USA

**Keywords:** Sulfolane, Rat, Mice, Guinea pig, Kidney, Plasma concentration

## Abstract

Sulfolane is a solvent used in industrial refining with identified environmental exposure in drinking water. Due to potential large species differences, the National Toxicology Program (NTP) conducted 28-day toxicity studies in male and female Hsd:Sprague Dawley® SD® rats, B6C3F1/N mice, and Hartley guinea pigs. A wide dose range of 0, 1, 10, 30, 100, 300, and 800 mg/kg was administered via gavage. Histopathology, clinical pathology, and organ weights were evaluated after 28 days of exposure. In addition, plasma concentrations of sulfolane were evaluated 2 and 24 h after the last dose. Increased mortality was observed in the highest dose group of guinea pigs and mice while decreased body weight was observed in rats compared to controls. Histopathological lesions were observed in the kidney (male rat), stomach (male mice), esophagus (male and female guinea pigs), and nose (male guinea pigs). Plasma concentrations were generally higher in rats and guinea pigs compared to mice with evidence of saturated clearance at higher doses. Male rats appear to be the most sensitive with hyaline droplet accumulation occurring at all doses, although the human relevance of this finding is questionable.

## Highlights

• Sulfolane was evaluated in mice, rat, and guinea pig 28-day toxicity studies.
• The rat was more sensitive compared to mice and guinea pigs.
• Rats generally had the highest plasma concentrations at 24 h after the last dose.

## Graphical abstract

**Figure:**
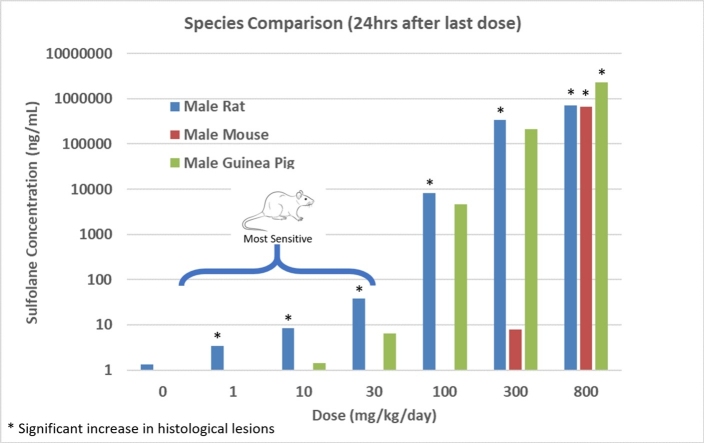

## 1 Introduction

Sulfolane, or tetrahydrothiophene 1,1-dioxide, is a highly polar, organosulfur compound used as a solvent in liquid-liquid and liquid-vapor extraction processes common during industrial refining; e.g. extraction of aromatic hydrocarbons from petroleum [1,2,3]. It was originally developed by Shell Oil Company in the 1950s as a solvent for purification of butadiene [4]. Additional industrial uses for sulfolane include the fractionalization of wood tars, the manufacturing of electronics and polymers, and as a curing agent in epoxy resins [3]. In 2010, it was estimated that approximately 150 extraction units utilizing sulfolane are in use across the world, and sulfolane is considered a high production volume (HPV) chemical in the United States; in 2006, the latest date for which sulfolane production information is available, annual U.S. production was between 10–50 million pounds [5,6].

Sulfolane is produced via a reaction between sulfur dioxide and butadiene, which forms sulfolene; sulfolene is then hydrogenated to form sulfolane. Sulfolane has a low vapor pressure (0.0062 mm Hg at 27.6 °C), no odor, and is miscible in water, acetone, glycerol, and numerous oils; it has a viscosity of 10.34 centipoises at 30 °C [1,7]. It is presumed to not break down easily in groundwater, likely due to low oxygen and nutrient levels, and, while sulfolane does not accumulate in the aquatic food chain, it is taken up by plants [8,9].

Human exposure to sulfolane can occur in occupational (inhalation and/or dermal) and environmental (drinking water) settings; it has been detected in groundwater sources near refining sites. The groundwater in North Pole, Alaska is known to be contaminated with sulfolane from a nearby petroleum refinery. It has been detected in nearly 300 drinking water wells in the area since 2009, with measurements currently ranging between 4–7 parts per billion (ppb) in older supply wells [10]. In addition to North Pole, AK, sulfolane has also been detected at additional sites in Canada and the United States near areas of natural gas or petroleum refining. There are currently no federal regulatory limits for sulfolane levels in drinking water; however, ATSDR’s recommended public health action levels in drinking water (as ppb) for sulfolane based on guinea pig studies are 20 (infants), 32 (children), and 70 (adults) based on average water intake [11]. EPA’s Provisional Peer-Reviewed Toxicity Values (PPRTV) for subchronic and chronic sulfolane exposure are 10 μg/kg/day and 1 μg/kg/day, respectively, based on decreased white blood cell counts in female rats after drinking water exposure for 90 days [12].

Sulfolane is known to be well-absorbed following intravenous and oral administration in rats, but not following dermal exposure in humans [2,13,14]. Studies of the absorption, distribution, metabolism, and excretion (ADME) of sulfolane, while limited, suggest a short half-life (3.5–5 hours in rabbits, dogs, and monkeys) and a large volume of distribution following intravenous administration [2]. A wide tissue distribution, including the brain, and maternal transfer during gestation have also been reported following oral exposure [15]. The primary metabolite of sulfolane in rats, mice, and rabbits is 3-hydroxysulfolane, which is believed to be primarily responsible for sulfolane’s reported effects on thermoregulation [16,17]. Recent comparative studies in mice and rats showed no apparent sex differences and a half-life of 2–6 hours in rats depending on dose and ≤ 1.3 h in mice [18], with poor dermal absorption in rats, but not in mice [19].

Sulfolane has not been shown to be a dermal irritant or a sensitizer. However, indications of central nervous system toxicity, including convulsions, seizures, and hyper/hypoactivity, have been noted in rodents following high inhalation exposures to sulfolane and have also been reported in additional acute toxicity studies following intraperitoneal exposure [2,20,21]. Gordon et al. [22,23] reported similar acute neurotoxic effects in rats following sulfolane exposure that included alterations in motor activity and brain-wave patterns. Sulfolane induces regulated hypothermia in mice, and the toxicity and lethality of sulfolane in animal models is directly correlated with decreases in ambient body temperature [21,24].

In subchronic inhalation studies of aerosolized sulfolane (3% water) conducted in rats, guinea pigs, dogs, and squirrel monkeys, chronic lung inflammation was observed in all species following 27 days of exposure (8 h/day, 5 days/week) to 495 mg/m^3^ sulfolane; chronic liver inflammation was also observed in rats, and mortality occurred in monkeys [20]. In a subchronic study, decreased white blood cell counts and increased incidences of fatty liver occurred in guinea pigs exposed to 200 mg/m^3^ sulfolane for approximately 90 days (23 h/day, 7 days/week). Mortality occurred in dogs and monkeys exposed to the same dose (200 mg/m^3^) for the same period of time, and a no-observed-adverse-effect-level (NOAEL) of 20 mg/m^3^ was calculated by the authors for all species [20].

In a 28-day toxicity study, male and female rats exposed to 700 mg/kg sulfolane via oral gavage experienced weight loss and decreased food consumption; female rats recovered after the second week of exposure [25]. Increased incidences of hyaline droplet accumulation in the kidneys (males) and decreased erythrocyte counts and spleen weights (females) were also observed [25]. In a 90-day drinking water study of sulfolane (0, 25, 100, 400, and 1600 mg/L), a lowest-observed-adverse-effect-level (LOAEL) and a NOAEL of 100 mg/L (10.6 mg/kg/day) and 25 mg/L (2.9 mg/kg/day), respectively, were reported for female rats based on decreased white blood cells (WBC), lymphocytes, monocytes, basophils, and large unstained cells (LUCs) at higher doses [26]. In 90-day gavage studies (0, 55.6, 167, and 500 mg/kg), clinical chemistry changes were reported in rats and guinea pigs in addition to decreased WBC counts in guinea pigs [15]. In a second study in guinea pigs orally exposed for six months (0, 0.25, 2.5, 25, 250 mg/kg), clinical pathology changes, lesions in the liver and spleen, and effects on the WBC were reported [15].

Reproductive effects have been reported in rats following exposure to sulfolane via oral gavage, including reduced estrous cycles, increased litter loss, decreased pup number, and low pup weight [25,27]. Fetal resorptions and some fetal skeletal abnormalities were noted in a prenatal developmental toxicity study of sulfolane in mice [15]. In 2-year carcinogenicity studies of 3-sulfolene, an intermediate in the production of sulfolane, mortality was noted in both rats and mice, but there was no evidence of carcinogenic activity [28,29]. There are currently no human data on the possible health effects following oral sulfolane exposure.

Common findings in the literature suggest sulfolane exposure leads to effects on the kidney, spleen, and white blood cells in rats and guinea pigs. Based on this information, ATSDR and EPA have provided health guidance using data from different species. Limited data from studies in the literature suggest that the guinea pig model may be more sensitive to sulfolane compared to rats, while minimal information is available in mice. The goal of our studies presented here is to provide a direct comparison of effects across these three species and provide data for comparisons of internal dose, which would help provide guidance for exposure to this chemical. In order to characterize the potential species and sex differences of sulfolane toxicity, the National Toxicology Program (NTP) conducted 28-day repeat dose toxicity studies in male and female Sprague Dawley rats, B6C3F1/N mice, and Hartley guinea pigs exposed via oral gavage. As part of this assessment, plasma concentrations of sulfolane were measured in each species and sex to provide information on potential sex- and/or species-specific differences and aid in the interpretation of previous and future toxicity data.

## 2 Materials and methods

### 2.1 Chemicals

Sulfolane (CAS No. 126-33-0, Lot No. MKBH1265 V), was purchased from Aldrich (Milwaukee, WI). Infrared (IR) and nuclear magnetic resonance (NMR) spectra supported the identity of the lot as sulfolane and a chemical purity of >99.0% was determined by gas chromatography coupled with flame ionization detection. Deionized water was used as the vehicle for dose formulations. Sulfolane formulations in deionized water were stable for up to 42 days when stored in sealed clear glass bottles at ambient temperature. Formulations were analyzed both prior to and after administration to the animals. All pre- and post-administration values were within 10% of target concentrations except for one batch of post-administration where the value was within 14% of target.

### 2.2 Animals

Male and female Sprague Dawley (Hsd:Sprague Dawley®SD®) rats were obtained from Harlan Laboratories, Inc. (now Envigo) (Indianapolis, IN) at 27–33 days of age (39–46 days of age at start of dosing). Male and female B6C3F1/N mice were obtained from Taconic Biosciences, Inc. (Germantown, NY) at 24–30 days of age (36–43 days of age at start of dosing). Male and female Hartley guinea pigs, 24–30 days of age (36–43 days of age at start), were obtained from Charles River Laboratories (Wilmington, MA) from the Saint Constant, Quebec, Canada facility. All animal studies were conducted in an animal facility accredited by the Association for the Assessment and Accreditation of Laboratory Animal Care International. Studies were approved by the Battelle Animal Care and Use Committee and conducted in accordance with all relevant NIH and NTP animal care and use policies and applicable federal, state, and local regulations and guidelines.

Irradiated NTP-2000 wafer feed, supplied by Zeigler Brothers, Inc. (Gardners, PA), was provided *ad libitum* in hanging stainless-steel feeders (Lab Products, Inc.) to rats and mice. Irradiated RQ 18−4 pelleted feed, supplied by Zeigler Brothers, Inc. (Gardners, PA), was provided *ad libitum* in bowls or J-feeders (Lab Products, Inc.) to guinea pigs. Water from the City of Columbus (Ohio) municipal supply was provided on an *ad libitum* basis via the automatic rack watering system (Edstrom Industries, Inc., Waterford, WI) without further treatment. Animals were housed in solid polycarbonate cages manufactured by Lab Products, Inc. (Seaford, DE). Guinea pigs were group housed up to two per cage, rats and mice up to five per cage. Bedding for the cages consisted of irradiated Sani-Chips® hardwood chips (P.J. Murphy Forest Products Corporation, Montville, NJ).

### 2.3 Sentinel animals

Blood (serum) for serological analysis was collected from five male and female rats, mice, or guinea pigs and submitted to IDEXX BioResearch (Columbia, MO) and analyzed; no positive titers were detected in rats or mice. Ten guinea pig samples collected prior to quarantine release tested positive for CavPI3 (parainfluenza virus). The positive CavPI3 results were interpreted to be subclinical by the attending staff veterinarian, having no adverse impact on the study. Cecal contents were collected and no internal or external parasites were detected in rats, mice, or guinea pigs.

### 2.4 Study design

Randomization (stratified by body weight) to dosage groups was performed using NTP Provantis software (version 9.2.3) (Instem, Stone, UK).

Sulfolane in deionized water was administered once a day by oral gavage to male and female Sprague Dawley rats, B6C3F1/N mice, and Hartley guinea pigs (0, 1, 10, 30, 100, 300, and 800 mg/kg). A wide range of doses was selected due to unknown species sensitivities, and challenged with a high dose of 800 mg/kg consistent with a previously reported rat study [25]. Formulations were administered to rats and guinea pigs at a volume of 5 mL/kg, to mice at a volume of 10 mL/kg, and were based on each animal’s most recent body weight. In order to prevent bias in the time of treatment, dose administration began with a different dosage group each day. All animals were dosed for 28 consecutive days. The core group for evaluation consisted of 10 animals/sex/species, except for the guinea pig control group, which contained 20 animals/sex/species to increase control confidence. Plasma concentrations were measured at 2 and 24 h after the last dose to assess potential species differences following dosing and at necropsy. To determine plasma chemical concentrations, additional rats, mice, and guinea pigs (special study groups; n = 3 animals/sex/species) were included for sample analysis at 2 h in selected dose groups (0, 30, 100, 300 mg/kg). Additionally, extra mice (n = 3 animals/sex/species) were added to all dosage groups for sample analysis at 24 h after the last dose due to concern of inadequate blood volume.

Animals were observed for signs of mortality or moribundity and other clinical signs. Animals were weighed prior to dosing on day 1, twice weekly thereafter (except mice were weighed only on days 1 and 8 for the first week), and at study termination. Rectal temperatures were recorded on day 28 (special study animals) and day 29 (core animals), as well as on the day of euthanasia for animals that were euthanized early.

### 2.5 Blood collection

On day 28, 2 h following the last dose administration, special study animals were anesthetized with CO_2_/O_2_ (approximately 70/30%) and blood was collected, using K_3_ EDTA as an anticoagulant, from the retro-orbital plexus (rats), retro-orbital sinus (mice), or cranial vena cava (guinea pigs) of the 0, 30, 100, and 300 mg/kg special study groups for determination of plasma sulfolane concentrations. On day 29, 24 h after the last dose administration, blood samples were collected from the remaining special study mice for determination of plasma sulfolane concentrations and from all surviving core study animals for plasma sulfolane concentrations, hematology, clinical chemistry (rats and guinea pigs only), and micronuclei determinations following anesthesia with CO_2_/O_2_ (approximately 70/30%). Blood was stored on ice until plasma was isolated. Plasma was stored at −70 °C. No animals were fasted prior to blood collection.

#### 2.5.1 Analysis of sulfolane in plasma

Sulfolane concentrations in plasma were determined using a validated gas chromatography-mass spectrometry (GC–MS) method using an Agilent 7890A gas chromatograph coupled to a 5975C mass selective detector (Agilent Technologies, Santa Clara, CA) [49]. Briefly, to 100 μL of plasma, 50 μL 1 N NaOH was added along with 10 μL of d_8_-sulfolane (internal standard) in deionized water (2 μg/mL or 200 μg/mL, depending on the anticipated sulfolane concentration in sample). Samples were extracted with 0.5 mL ethyl acetate and the supernatants were analyzed by GC–MS. Study samples that exceeded the calibration curve range were diluted into the validated analytical range using extracted respective control matrix. Each sample set was run with calibration standards and bracketed by quality control (QC) samples prepared at low and high ends of the respective calibration curve. A linear regression with 1/x^2^ weighing was used to relate GC–MS peak area response ratio of analyte to internal standard and concentration of sulfolane in plasma. The concentration of sulfolane in samples was calculated using response ratio, regression equation, plasma volume, and dilution when applicable. The concentration of analytes was expressed as ng/mL of plasma. All concentrations above the LOD (1.25 ng/mL) of the assay were reported. Data from study samples were considered valid if: the matrix calibration curve was linear (r ≥ 0.99); matrix standards were within 10% of nominal; at least 67% of the QC samples were within 15% of nominal values. All QCs were within 15% of nominal value.

#### 2.5.2 Hematology

Blood for hematologic assessment was collected in K_3_ EDTA tubes. The following parameters were measured using an ADVIA 120 hematology analyzer (Bayer Diagnostics Division, Tarrytown, NY): hemoglobin concentration, hematocrit, mean cell volume (MCV), mean cell hemoglobin (MCH), mean cell hemoglobin concentration (MCHC), white blood cell (WBC) count and differential, erythrocyte count, reticulocyte count, platelet count. A spun (manual) hematocrit was also calculated. Peripheral blood smears were evaluated for any abnormal cellular morphologies.

#### 2.5.3 Clinical chemistry

Blood was collected into a serum separator tube for clinical chemistry assessment and allowed to clot for at least 30 min. The tube was then centrifuged and the serum collected. The following clinical chemistry parameters were analyzed using the Cobas c501 Chemistry Analyzer (Roche, Indianapolis, IN): total protein, albumin, urea nitrogen, creatinine, alanine aminotransferase (ALT), sorbitol dehydrogenase (SDH), alkaline phosphatase, total bile acids, glucose, creatine kinase, cholesterol, and triglycerides.

#### 2.5.4 Micronuclei determination

Micronucleus frequencies were not measured in guinea pigs due to the lack of historical control data as well as the unavailability of internal standards for flow cytometric analysis of blood samples. For rats and mice, blood samples (200 μL per animal) were collected at necropsy and stabilized in EDTA tubes. For each sample, 50 μL of blood were dispensed into a microcentrifuge tube containing heparin and mixed by inverting several times. A fixation tube containing ultra-cold methanol was then removed from a −80 °C freezer and 180 μL of the heparinized blood sample was forcefully dispensed into the tube, rapidly mixed, and quickly transferred back to the −80 °C freezer. This process was repeated for each blood sample. The fixed blood samples were stored in the −80 °C freezer for at least three days prior to flow cytometry analysis of micronucleated red blood cells.

Flow cytometry analysis was performed using MicroFlow^PLUS^ Kit reagents (Litron Laboratories, Rochester, NY) and a Becton-Dickinson FACSCalibur™ dual-laser bench top flow cytometry system (Becton Dickinson Biosciences, San Jose, CA). The analysis was performed according to the kit’s instructional manual with minimal modification [31,50]. For 5–6 peripheral blood samples/dose group, 20,000 (± 2000) immature CD71-positive erythrocytes (also referred to as reticulocytes) were analyzed to determine the frequency of normal (RET) and micronucleated RET (MN-RET). Normal and micronucleated mature erythrocytes (MN—E) (CD71-negative) were enumerated concurrently during MN-RET analysis. Aggregates were excluded on the basis of forward and side scatter, platelets were excluded based on staining with an anti-CD61 antibody, and nucleated leukocytes were excluded on the basis of intense propidium iodide staining. Numbers of mature erythrocytes evaluated in rats totaled from 1.2 × 10^5^ – 3.2 × 10^6^ (males) and 1.2 – 3.9 × 10^6^ (females) cells, and in mice, totaled generally from 1.1 to 2.3 × 10^6^ (males) and 7.9 × 10^5^ – 1.6 × 10^6^ (females) cells, allowing for calculation of the percentage of RET among total erythrocytes as a measure of bone marrow toxicity. For the rat samples, only RETs with the highest CD71 activity were evaluated due to the speed and efficiency with which the rat spleen removes damaged RETs from circulation. Thus, although micronucleus frequency was evaluated in both immature and mature erythrocytes, the appropriate cell population for this assessment in rats is the young RET population.

### 2.6 Necropsy

At the final scheduled termination, organ weights were collected for the liver, thymus, spleen, left and right kidney, left and right testis, left and right epididymis, left and right ovary, heart, and lungs. Bilateral organs were weighed separately. At necropsy, tissues were collected (see NTP Specifications) and fixed in 10% neutral buffered formalin (NBF) except for the eyes and testes with epididymides/vaginal tunics, which were initially placed in Davidson’s solution and modified Davidson’s solution, respectively, and then transferred to 10% NBF.

### 2.7 Statistical analysis

Group mean body weights, organ weight data, and microscopic pathology data were collected and summarized using the NTP Provantis system (version 9.2.3); group mean hematology data were collected and summarized using the Battelle Provantis system (version 8.6.1.2).

Two approaches were employed to assess the significance of pairwise comparisons between dosed and control groups in the analysis of continuous variables. Organ and body weight data, which historically have approximately normal distributions, were analyzed with the parametric multiple comparison procedures of Dunnett [33] and Williams [34,35]. Hematology and clinical chemistry data, which have typically skewed distributions, were analyzed using the nonparametric multiple comparison methods of Shirley [36] (as modified by Williams [37] and Dunn [38]). Jonckheere’s test [51] was used to assess the significance of the dose-related trends and to determine whether a trend-sensitive test (Williams’ or Shirley’s test) was more appropriate for pairwise comparisons than a test that does not assume a monotonic dose-related trend (Dunnett’s or Dunn’s test). Prior to statistical analysis, extreme values identified by the outlier test of Dixon and Massey [40] were examined by NTP personnel, and implausible values were eliminated from the analysis.

The incidence of histopathological lesions is presented as the number of animals examined for each organ along with the number of animals with observation reported as percent incidence. Systemic lesions were included once in the total lesion count regardless of how many times they occurred in a single animal. The Fisher exact test [41] was used to determine significance.

Based on the large number of cells evaluated using flow cytometric techniques [42], it is assumed that the proportion of micronucleated cells is normally distributed. The appropriate statistical tests for trend and for pairwise comparisons with the control group depend on whether the variances among the groups are equal. Levene’s test at α = 0.05 is used to test for equal variances. When variances are equal, linear regression is used to test for a linear trend with dose, and Williams’ test is used to test for pairwise differences between each treatment group and the control group. When variances are unequal, Jonckheere’s test is used for linear trend and Dunn’s test for pairwise comparisons of each treatment group with the control group. To correct for the multiple pairwise comparisons, the *P* value for each comparison with the control group is multiplied by the number of comparisons made (in the case of sulfolane, this number was 4). When this product is greater than 1.00, it is replaced with 1.00. Statistical significance for all tests was set at *P* ≤ 0.025. Mean MN-RET/1000 RET and MN-E/1000 erythrocytes, as well as %RET, were calculated for each animal. These data are summarized in the tables as mean ± standard error of the mean. In the micronucleus assay, a positive response is preferably based on the observation of both a significant trend as well an observation of at least one dose group significantly elevated over the concurrent control group. If only one statistical test (trend or pairwise) is significant, the micronucleus assay is judged to be equivocal. The absence of both a significant trend and a significant dose results in a negative call for the assay.

## 3 Results

All data from the 28-day toxicity studies of sulfolane are available in the National Toxicology Program (NTP) Chemical Effects in Biological Systems (CEBS) database: https://doi.org/10.22427/NTP-DATA-002-03276-0000-0000-0. Data not shown here are available as supplemental data in CEBS.

### 3.1 Survival

All rats survived to scheduled termination. Mortality occurred in both male and female mice administered 800 mg/kg sulfolane beginning on day 10 and day 6, respectively. In response, early euthanasia of mice in the 800 mg/kg group occurred. Total mortality in the 800 mg/kg group was 46% (6/13) for males and 62% (8/13) for females. An immediate cause of death was not apparent, with no clinical observations or gross lesions associated with mortality. All male and female mice in the remaining 0, 1, 10, 30, 100, and 300 mg/kg groups survived to their scheduled termination on days 28 and 29. Mortality also occurred in both male and female guinea pigs administered 800 mg/kg sulfolane beginning on day 3. In response to high dose mortality, early euthanasia of guinea pigs in the 800 mg/kg group occurred. All male and female guinea pigs in all other dosage groups survived to study termination.

### 3.2 Clinical observations

Clinical signs of toxicity attributed to sulfolane administration consisted of hunched posture, cold to the touch, and ruffled coat in both male and female rats in the 800 mg/kg groups; one 800 mg/kg female rat exhibited seizure activity. Clinical signs of toxicity in guinea pigs were limited to the 800 mg/kg dose group and included males observed as lethargic or with seizures and females observed as hyperactive.

### 3.3 Body weights

**Fig. 1:**
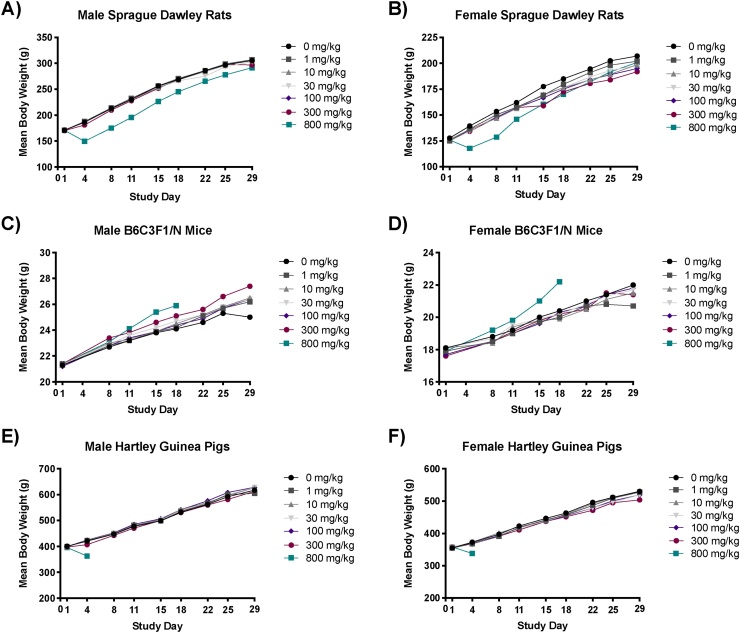
**Mean body weight of male and female Sprague Dawley rats, B6C3F1/N mice, and Hartley guinea pigs exposed to sulfolane for 28 days.** A-B) Mean body weight of male and female Sprague Dawley rats. C-D) Mean body weight of male and female B6C3F1/N mice. E-F) Mean body weight of male and female Hartley guinea pigs. Data presented as mean.

Group mean body weights of 800 mg/kg male rats were lower than those of vehicle control (up to -20%) early in the study, but these reductions became less pronounced over time (Fig. 1A). Female rats displayed a similar pattern of lower mean body weights early in the study, but appeared to be more sensitive compared to males (Fig. 1B). Group mean body weights of male and female mice were similar to their respective vehicle groups with the exception of the 800 mg/kg dose group, which were increased (8–9%) relative to their respective vehicle control (Fig. 1C and D) prior to termination. Group mean body weights of guinea pigs were decreased in males (-14%) and females (-9.3%) administered 800 mg/kg sulfolane (Fig. 1E and F).

### 3.4 Organ weights

Absolute kidney weights were increased in the top two dose groups (up to 15–16% compared to control) in addition to relative weights in male rats. (Table 1). In female rats, absolute and relative kidney weights were significantly increased in the 800 mg/kg group (Table 1). There were no treatment-related changes in kidney weights in male or female guinea pigs (supplemental data).

**Table 1:** Terminal body weight and selected absolute and relative organ weights of male and female Sprague Dawley rats after 28-day repeat dose exposure to sulfolane^a^.

Species/Sex	Weights	0 mg/kg	1 mg/kg	10 mg/kg	30 mg/kg	100 mg/kg	300 mg/kg	800 mg/kg
Male Rats	Body (g)	305.3 ± 8.2*	307.1 ± 5.3	306.7 ± 4.2	303.0 ± 5.6	306.2 ± 6.0	296.8 ± 6.5	291.6 ± 3.9
	Liver (Absolute) (g)	13.08 ± 0.52**	13.23 ± 0.27	13.63 ± 0.41	13.45 ± 0.35	13.43 ± 0.39	14.46 ± 0.46*	15.92 ± 0.45**
	Liver (Relative) (mg/g)	42.78 ± 1.09**	43.14 ± 0.91	44.46 ± 1.26	44.37 ± 0.72	43.87 ± 0.88	48.72 ± 1.10**	54.56 ± 1.07**
	Right Kidney (Absolute) (g)	1.06 ± 0.04**	1.11 ± 0.03	1.11 ± 0.02	1.13 ± 0.03	1.11 ± 0.03	1.19 ± 0.03**	1.22 ± 0.03**
	Right Kidney (Relative) (mg/g)	3.48 ± 0.08**	3.60 ± 0.10	3.62 ± 0.07	3.73 ± 0.05	3.61 ± 0.05	4.03 ± 0.06**	4.19 ± 0.09**
	Left Kidney (Absolute) (g)	1.04 ± 0.04**	1.08 ± 0.02	1.10 ± 0.02	1.09 ± 0.03	1.10 ± 0.02	1.20 ± 0.03**	1.21 ± 0.03**
	Left Kidney (Relative) (mg/g)	3.41 ± 0.08**	3.52 ± 0.06	3.59 ± 0.07	3.58 ± 0.07	3.60 ± 0.04	4.04 ± 0.07**	4.16 ± 0.10**
	Spleen (Absolute) (g)	0.664 ± 0.037*	0.673 ± 0.019	0.701 ± 0.028	0.714 ± 0.026	0.694 ± 0.027	0.639 ± 0.022	0.575 ± 0.022
	Spleen (Relative) (mg/g)	2.17 ± 0.09*	2.20 ± 0.06	2.28 ± 0.08	2.36 ± 0.09	2.26 ± 0.07	2.15 ± 0.05	1.97 ± 0.06
	Lung (Absolute) (g)	2.14 ± 0.19	2.09 ± 0.09	2.01 ± 0.10	2.08 ± 0.12	2.18 ± 0.10	2.31 ± 0.10	2.21 ± 0.20
	Lung (Relative) (mg/g)	6.96 ± 0.50*	6.81 ± 0.32	6.55 ± 0.30	6.85 ± 0.37	7.11 ± 0.21	7.79 ± 0.30	7.62 ± 0.70
Female Rats	Body (g)	207.0 ± 4.4	202.0 ± 4.6	198.7 ± 4.4	197.9 ± 3.7	194.9 ± 3.9	191.9 ± 4.2	200.9 ± 3.4
	Liver (Absolute) (g)	8.30 ± 0.29	8.29 ± 0.28	7.91 ± 0.27	7.98 ± 0.23	7.92 ± 0.30	8.39 ± 0.27	9.53 ± 0.39*
	Liver (Relative) (mg/g)	40.03 ± 0.79**	40.99 ± 0.89	39.78 ± 0.87	40.29 ± 0.65	40.54 ± 0.89	43.65 ± 0.84*	47.35 ± 1.53**
	Right Kidney (Absolute) (g)	0.71 ± 0.02*	0.72 ± 0.02	0.66 ± 0.01	0.71 ± 0.02	0.68 ± 0.01	0.72 ± 0.02	0.79 ± 0.02**
	Right Kidney (Relative) (mg/g)	3.43 ± 0.06**	3.57 ± 0.04	3.35 ± 0.06	3.58 ± 0.06	3.48 ± 0.05	3.76 ± 0.05**	3.93 ± 0.05**
	Left Kidney (Absolute) (g)	0.70 ± 0.02*	0.72 ± 0.02	0.65 ± 0.01	0.69 ± 0.02	0.68 ± 0.01	0.71 ± 0.02	0.77 ± 0.02*
	Left Kidney (Relative) (mg/g)	3.37 ± 0.06**	3.54 ± 0.06	3.29 ± 0.05	3.47 ± 0.05	3.48 ± 0.07	3.72 ± 0.07**	3.85 ± 0.06**
	Spleen (Absolute) (g)	0.548 ± 0.024*	0.554 ± 0.019	0.528 ± 0.014	0.524 ± 0.017	0.553 ± 0.024	0.522 ± 0.018	0.473 ± 0.013*
	Spleen (Relative) (mg/g)	2.65 ± 0.11	2.74 ± 0.05	2.66 ± 0.07	2.64 ± 0.05	2.84 ± 0.12	2.71 ± 0.04	2.35 ± 0.05*
	Lung (Absolute) (g)	1.66 ± 0.13**	1.64 ± 0.08	1.54 ± 0.06	1.55 ± 0.07	1.53 ± 0.02	1.75 ± 0.07	1.96 ± 0.07**
	Lung (Relative) (mg/g)	8.01 ± 0.53**	8.10 ± 0.33	7.75 ± 0.24	7.85 ± 0.39	7.90 ± 0.17	9.12 ± 0.28*	9.79 ± 0.35**

a Data are displayed as mean ± SEM. Relative organ weights (organ-weight-to-body-weight ratios) are given as mg organ weight/g body weight. Statistical analysis performed by Jonckheere (trend) and Williams or Dunnett (pairwise) tests. Statistical significance for the control group indicates a significant trend test. Statistical significance for a treatment group indicates a significant pairwise test compared to the vehicle control group.

* Statistically significant at P ≤ 0.05.

** Statistically significant at P ≤ 0.01.

Absolute and relative liver weights were increased in 300 and 800 mg/kg male rats (Table 1). Relative liver weights were significantly increased in 300 mg/kg female rats, while both absolute and relative liver weights were increased in 800 mg/kg female rats (Table 1). In male mice, absolute liver weights were significantly increased (+12.4%) in the 300 mg/kg group, while relative liver weight was increased in 300 mg/kg female mice. A significant positive trend in liver weight (both absolute and relative) was observed in both sexes of mice (Table 2). The observed increases in liver weight in both rats and mice were not associated with any abnormal microscopic lesions. There were no treatment-related changes in liver weight or histopathology in male or female guinea pigs (Table 2).

**Table 2:** Terminal body weight and selected absolute and relative organ weights of male and female B6C3F1/N mice and Hartley guinea pigs after 28-day repeat dose exposure to sulfolane^a^.

Species/Sex	Weights	0 mg/kg	1 mg/kg	10 mg/kg	30 mg/kg	100 mg/kg	300 mg/kg	800 mg/kg
Male Mice	Body (g)	25.4 ± 0.6*	25.9 ± 0.5	26.4 ± 0.4	26.4 ± 0.7	26.4 ± 0.3	27.3 ± 0.4*	–
	Liver (Absolute) (g)	1.29 ± 0.05**	1.33 ± 0.06	1.38 ± 0.02	1.37 ± 0.06	1.41 ± 0.02	1.45 ± 0.03*	–
	Liver (Relative) (mg/g)	50.80 ± 0.87*	51.27 ± 1.48	52.41 ± 0.65	51.98 ± 1.32	53.56 ± 0.77	53.03 ± 0.93	–

Female Mice	Body (g)	22.1 ± 0.2	21.0 ± 0.5	21.5 ± 0.4	21.8 ± 0.4	21.7 ± 0.2	21.8 ± 0.3	–
	Liver (Absolute) (g)	1.10 ± 0.03*	1.09 ± 0.03	1.12 ± 0.04	1.09 ± 0.02	1.13 ± 0.02	1.16 ± 0.02	–
	Liver (Relative) (mg/g)	49.78 ± 1.02*	51.85 ± 0.59	51.87 ± 1.05	49.95 ± 0.65	52.23 ± 0.65	53.09 ± 0.62*	–

Male Guinea Pigs	Body (g)	617.7 ± 9.9	604.4 ± 9.2	626.7 ± 9.8	628.3 ± 9.9	628.6 ± 7.1	613.1 ± 13.2	–
	Liver (Absolute) (g)	24.20 ± 1.05	23.28 ± 1.05	23.21 ± 0.99	24.56 ± 1.24	25.65 ± 1.22	22.12 ± 0.94	–
	Liver (Relative) (mg/g)	38.94 ± 1.18	38.39 ± 1.25	36.98 ± 1.27	38.98 ± 1.58	40.82 ± 1.92	36.04 ± 1.20	–

Female Guinea Pigs	Body (g)	529.7 ± 11.4	527.5 ± 11.4	531.2 ± 10.7	519.5 ± 6.9	519.0 ± 8.5	503.9 ± 7.5	–
	Liver (Absolute) (g)	19.77 ± 0.86	18.98 ± 0.80	20.04 ± 1.08	18.16 ± 1.03	19.13 ± 0.42	17.76 ± 0.88	–
	Liver (Relative) (mg/g)	37.06 ± 1.00	35.86 ± 0.90	37.54 ± 1.42	34.85 ± 1.68	36.89 ± 0.79	35.18 ± 1.45	–

Relative organ weights (organ-weight-to-body-weight ratios) are given as mg organ weight/g body weight. Statistical analysis performed by Jonckheere (trend) and Williams or Dunnett (pairwise) tests. Statistical significance for the control group indicates a significant trend test. Statistical significance for a treatment group indicates a significant pairwise test compared to the vehicle control group.

a Data are displayed as mean ± SEM.

* Statistically significant at *P* ≤ 0.05.

** Statistically significant at *P* ≤ 0.01.

Absolute and relative spleen weights were significantly decreased in female rats in the 800 mg/kg dose group (Table 1); Relative lung weights were significantly increased in female rats in the 300 mg/kg group, while both absolute and relative lung weights were increased in female rats in the 800 mg/kg group (Table 1). The decrease in spleen weight and increase in lung weight did not have a microscopic correlate.

### 3.5 Rectal temperature

Rectal temperatures of 800 mg/kg sulfolane-treated male and female rats were lower than their respective vehicle groups. Due to early mortality, rectal temperatures were only collected from seven 800 mg/kg male mice and five 800 mg/kg female mice with mean temperatures of 95.3 and 95.9 °F, respectively; these temperatures were lower than the normal range of 97.7–100.4 °F while the rest of the groups were similar to their respective vehicle group. Rectal temperatures of all sulfolane-treated male and female guinea pigs were similar to their respective vehicle groups (supplemental data).

### 3.6 Hematology

MCV and MCH were increased in the 300 mg/kg (4% and 3%, respectively) and 800 mg/kg (6% and 5%, respectively) male rats, and the reticulocyte count also increased (35%) in the 800 mg/kg male rats (Table 3). In female rats, the MCV was increased (3%) in the 800 mg/kg group. A negative trend, but no pair-wise differences, in erythrocyte counts was observed in both male and female rats.

**Table 3:** Selected hematology parameters in male and female Sprague Dawley rats, B6C3F1/N mice, and Hartley guinea pigs after 28-day repeat dose exposure to sulfolane^a^.

Species/Sex^b^	Endpoint	0 mg/kg	1 mg/kg	10 mg/kg	30 mg/kg	100 mg/kg	300 mg/kg	800 mg/kg
Male Rats	WBC (K/μL)	12.32 ± 0.67*	13.64 ± 0.74	13.85 ± 0.69	13.87 ± 0.67	13.12 ± 0.71	12.29 ± 0.79	10.98 ± 0.44
	Lymphocytes (K/μL)	10.38 ± 0.64**	11.17 ± 0.57	11.40 ± 0.62	11.46 ± 0.67	10.47 ± 0.58	9.92 ± 0.68	8.78 ± 0.37
	Reticulocytes (K/μL)	192.1 ± 5.6**	181.2 ± 4.2	190.7 ± 6.1	188.7 ± 8.3	194.9 ± 7.1	217.9 ± 11.9	259.3 ± 12.6**
	Erythrocytes (10^6^/μL)	8.15 ± 0.12*	8.17 ± 0.07	8.25 ± 0.10	8.05 ± 0.12	8.01 ± 0.11	8.23 ± 0.11	7.80 ± 0.08
	MCV (fL)	60.0 ± 0.6**	60.5 ± 0.6	59.7 ± 0.4	60.2 ± 0.5	60.5 ± 0.5	62.1 ± 0.3**	63.3 ± 0.4**
	MCH (pg)	18.6 ± 0.2**	18.7 ± 0.1	18.7 ± 0.1	18.8 ± 0.1	19.0 ± 0.2	19.2 ± 0.1**	19.5 ± 0.1**
Female Rats	WBC (K/μL)	12.05 ± 0.93*	10.96 ± 0.66	10.69 ± 0.61	10.74 ± 0.45	11.15 ± 0.97	10.15 ± 0.69	9.37 ± 0.52
	Lymphocytes (K/μL)	10.06 ± 0.81*	8.89 ± 0.58	8.99 ± 0.60	9.23 ± 0.41	9.40 ± 0.87	8.66 ± 0.60	7.74 ± 0.44
	Reticulocytes (K/μL)	199.8 ± 15.7	183.2 ± 10.0	171 ± 12.5	176.5 ± 10.4	178.8 ± 10.8	200.5 ± 17.2	230.1 ± 14.7
	Erythrocytes (10^6^/μL)	7.84 ± 0.08*	7.75 ± 0.07	7.82 ± 0.11	7.88 ± 0.09	7.74 ± 0.12	7.73 ± 0.12	7.49 ± 0.10
	MCV (fL)	60.0 ± 0.5**	60.1 ± 0.5	59.3 ± 0.3	60.1 ± 0.3	59.4 ± 0.5	60.7 ± 0.6	61.8 ± 0.4*
	MCH (pg)	18.9 ± 0.2*	18.9 ± 0.1	18.8 ± 0.1	18.9 ± 0.1	18.9 ± 0.2	19.3 ± 0.2	19.3 ± 0.2
Male Mice	WBC (K/μL)	4.66 ± 0.26	5.08 ± 0.35	4.70 ± 0.37	5.08 ± 0.28	5.94 ± 0.23*	5.06 ± 0.42	–
	Lymphocytes (K/μL)	3.86 ± 0.20	4.17 ± 0.28	3.87 ± 0.31	4.23 ± 0.22	4.83 ± 0.18*	4.16 ± 0.37	–
	Reticulocytes (K/μL)	269.3 ± 8.3	261.7 ± 8.0	267.4 ± 8.9	266.3 ± 7.7	269.8 ± 11.3	281.3 ± 4.9	–
	Erythrocytes (10^6^/μL)	10.83 ± 0.23**	10.76 ± 0.20	10.91 ± 0.16	10.82 ± 0.21	10.21 ± 0.14*	10.21 ± 0.12*	–
	MCV (fL)	46.7 ± 0.2**	46.8 ± 0.2	46.9 ± 0.1	46.9 ± 02	46.9 ± 0.2	47.9 ± 0.2**	–
Female Mice	WBC (K/μL)	5.32 ± 0.28	4.90 ± 0.31	4.65 ± 0.35	5.34 ± 0.41	5.04 ± 0.40	4.78 ± 0.32	–
	Lymphocytes (K/μL)	4.46 ± 0.23	4.19 ± 0.26	3.95 ± 0.30	4.54 ± 0.31	4.22 ± 0.36	3.82 ± 0.20	–
	Reticulocytes (K/μL)	254.4 ± 10.3	300.3 ± 9.4*	316.6 ± 12.5**	287.9 ± 12.9	291.2 ± 10.3	302.4 ± 12.9*	–
	Erythrocytes (10^6^/μL)	10.46 ± 0.09	11.04 ± 0.18	10.46 ± 0.13	10.5 ± 0.22	10.56 ± 0.17	10.55 ± 0.26	
	MCV (fL)	46.6 ± 0.2**	46.6 ± 0.2	46.9 ± 0.2	46.8 ± 0.2	46.9 ± 0.1	47.7 ± 0.2**	–
Male Guinea Pigs	WBC (K/μL)	6.49 ± 0.25	5.33 ± 0.28	5.70 ± 0.29	5.61 ± 0.35	5.90 ± 0.45	6.25 ± 0.15	–
	Lymphocytes (K/μL)	3.96 ± 0.15	3.19 ± 0.21*	3.26 ± 0.16*	3.40 ± 0.22	3.55 ± 0.29	3.38 ± 0.17	–
Female Guinea Pigs	WBC (K/μL)	5.81 ± 0.23	6.09 ± 0.33	5.59 ± 0.25	5.70 ± 0.34	6.18 ± 0.27	5.72 ± 0.23	–
	Lymphocytes (K/μL)	3.87 ± 0.17	3.94 ± 0.30	3.79 ± 0.21	3.80 ± 0.32	4.03 ± 0.20	3.99 ± 0.16	–

Statistical analysis performed by Jonckheere (trend) and Shirley or Dunn (pairwise) tests (unless otherwise noted).

Statistical significance for the control group indicates a significant trend test.

Statistical significance for a treatment group indicates a significant pairwise test compared to the vehicle control group.

WBC = white blood cells; MCV = mean cell volume; MCH = mean cell hemoglobin.

a Data are displayed as mean ± SEM.

b n = 9–10 (except guinea pig controls, where n = 19–20).

* Statistically significant at *P* ≤ 0.05.

** Statistically significant at *P* ≤ 0.01.

In the mice, mild decreases in the erythrocyte count (6%) were observed in the 100 and 300 mg/kg male mice with increases in the MCV observed in the male (3%) and female (3%) 300 mg/kg dosed groups (Table 3). In addition, reticulocyte counts were increased in the 1, 10, and 300 mg/kg female mice groups.

A decreasing trend in WBC and lymphocyte counts was observed in male and female rats; however, no pair-wise differences occurred (Table 3). Male mice in the 100 mg/kg group had an increase (∼25%) in the WBC and lymphocyte counts. In male guinea pigs, lymphocyte counts were decreased (∼18−20%) in the two low-dose groups (1 mg/kg and 10 mg/kg).

### 3.7 Clinical chemistry

ALT activity was mildly increased (43 %) in the high dose group (800 mg/kg) of both male and female rats (Table 4). SDH activity, another biomarker used for the detection of hepatocellular injury, was unchanged or decreased. Triglyceride concentrations were increased in the 800 mg/kg male rats. There were no clinical chemistry changes in male or female guinea pigs that could be attributed to the administration of sulfolane.

### 3.8 Histopathology

**Table 4:** Clinical chemistry findings in male and female Sprague Dawley rats after 28-day repeat dose exposure to sulfolane^a^.

Species/Sex^b^	Endpoint	0 mg/kg	1 mg/kg	10 mg/kg	30 mg/kg	100 mg/kg	300 mg/kg	800 mg/kg
Male Rats	Triglycerides (mg/dL)	78.1 ± 5.2**	83.8 ± 8.1	88.5 ± 7.0	111.4 ± 9.8	88.9 ± 7.4	75.5 ± 10.3	144.7 ± 11.9**
	ALT (IU/L)	52.20 ± 2.54**	50.00 ± 2.48	53.30 ± 1.22	50.70 ± 2.11	47.00 ± 1.81	55.10 ± 1.71	74.50 ± 1.54**
Female Rats	Triglycerides (mg/dL)	50.4 ± 4.6*	56.9 ± 4.6	67.7 ± 4.0	61.1 ± 7.4	56.7 ± 6.6	66.7 ± 6.9	90.6 ± 18.1
	ALT (IU/L)	49.40 ± 1.05**	46.80 ± 2.03	49.70 ± 3.26	48.40 ± 1.78	46.50 ± 1.28	52.00 ± 1.35	70.80 ± 3.00**

Statistical analysis performed by Jonckheere (trend) and Shirley or Dunn (pairwise) tests (unless otherwise noted).

Statistical significance for the control group indicates a significant trend test.

Statistical significance for a treatment group indicates a significant pairwise test compared to the vehicle control group.

ALT = alanine aminotransferase.

a Data are displayed as mean ± SEM.

b n = 10.

* Statistically significant at *P* ≤ 0.05.

** Statistically significant at *P* ≤ 0.01.

### 3.9 Kidney

Hyaline droplets were present in the cytoplasm of proximal renal tubular epithelial cells of male rats in every exposed group (Table 5). In general, the severity of the lesion (the number of cells affected and the size of the droplets) increased with dosage. In markedly affected animals, rare cortical tubular lumens contained a few fragmented, vacuolated cells. Tubular degeneration, which was minimal and uncommon, was nearly always present in only markedly hyaline droplet-affected animals and not diagnosed separately from hyaline droplet accumulation because it is considered to be related to the accumulation of hyaline droplets. Hyaline droplets were not observed in female rats, or male or female mice or guinea pigs, suggesting alpha 2U-globulin nephropathy. In males, accumulation of hyaline droplets within renal tubular epithelial cells was present in all dosage groups, thus the no-observed-effect level (NOEL) in males was 0 mg/kg. No histopathological effects were observed in the kidneys of male or female mice or guinea pigs.

**Table 5:** Histopathology findings in male and female Sprague Dawley rats, B6C3F1/N mice, and Hartley guinea pigs after 28-day repeat dose exposure to sulfolane^a^.

Species/Sex	0 mg/kg	1 mg/kg	10 mg/kg	30 mg/kg	100 mg/kg	300 mg/kg	800 mg/kg
Male Rats							
Kidney							
Hyaline Droplet Accumulation	0/10**	6/10** (1.0)	8/10** (1.0)	10/10** (1.1)	9/10** (1.1)	10/10** (2.6)	10/10** (3.1)
Male Mice							
Forestomach							
Hyperkeratosis	0/10	---	---	---	---	0/10	2/11 (1.0)
Glandular Stomach							
Glandular Hyperplasia; Chief Cell	0/10**	---	---	0/10	1/10 (1.0)	3/10 (1.0)	7/11** (1.0)
Mineral	0/10**	---	---	0/10	1/10 (1.0)	2/10 (1.0)	7/11** (1.0)
Male Guinea Pigs							
Esophagus							
Chronic Inflammation	1/20** (1.0)	2/2 (1.0)	---	1/1 (1.0)	1/1 (1.0)	1/10 (1.0)	5/10** (1.0)
Chronic Active Inflammation	0/20	0/2	---	0/1	0/1	0/10	1/10 (2.0)
Nose							
Chronic Active Inflammation	9/20* (1.7)	---	---	---	---	6/10 (1.7)	9/10** (1.6)
Female Guinea Pigs							
Esophagus							
Chronic Inflammation	0/20**	---	---	---	---	0/10	5/10** (1.0)

Mean severity grade denoted by: 1 – minimal; 2 – mild; 3 – moderate; 4 – marked.

Statistical analysis performed by Cochran-Armitage (trend) and Fisher Exact (pairwise) tests.

Statistical significance for the control group indicates a significant trend test. Statistical significance for a treatment group indicates a significant pairwise test compared to the vehicle control group.

All trend and pairwise p-values are reported as one-sided.

* Statistically significant at *P* ≤ 0.05.

** Statistically significant at *P* ≤ 0.01.

a Number of animals with observation reported with mean severity grade in parentheses.

### 3.10 Stomach

Doses greater than or equal to 100 mg/kg in male mice (Table 5) resulted in mineralization of gastric glandular epithelium (Table 5). Additionally, two female mice in the 800 mg/kg had mineralization of gastric glandular epithelium. Chief cell hyperplasia was evident in some males at doses greater than or equal to 300 mg/kg (Table 5). Hyperkeratosis of the stratified squamous epithelium in the forestomach was also observed in two mice from the 800 mg/kg dose group. Mineralization was present in the gastric fundus, extending to the limiting ridge in some animals. Affected epithelial cells were often located near the area of transition between chief cells and parietal cells (approximately 50–100 microns from the base of the gastric pit), with two 800 mg/kg males also having mineralization visible in the muscularis layer. Chief cell hyperplasia resulted in more intense basophilia in the depths of the gastric pits and extension of this alteration more superficially than is usually seen in the mucosa of vehicle animals. Cytoplasmic chief cell hyperplasia was always graded as minimal (grade 1). Mineralization of the gastric mucosa in the absence of evidence of renal damage is uncommon, but a similar lesion has been reported in rats administered gadolinium chloride intravenously [43]. No histopathological effects were observed in the gastric gland of male or female rats or guinea pigs.

### 3.11 Esophagus

Treatment-related chronic inflammation of the esophagus was observed in the high dose male and female guinea pigs. The inflammation was characterized by the presence of lymphocytes with fewer macrophages and rare multinucleated giant cells in the submucosa. In some animals, there was necrotic cell debris in the overlying mucosa, mainly in the keratin layer of the stratified squamous epithelium. The severity was minimal (grade 1) in all cases.

### 3.12 Nose

In the nose of the male guinea pigs, there was minimal to mild chronic active inflammation. The incidence in the 800 mg/kg dose group was statistically significant compared to the concurrent controls. The lesion was characterized by the presence of macrophages, lymphocytes, and fewer polymorphonuclear cells in the lamina propria and epithelium of levels I and II of the nasal cavity. The inflammation was noted in the respiratory and transitional epithelia and was minimal to mild in severity. Nasal inflammation was also observed in all female guinea pigs (controls and dose groups).

### 3.13 Plasma sulfolane concentration

Sulfolane concentrations were measured in plasma samples from males and females across the three species 2 and 24 h following the last dose administration (Table 6). For the 2 -h time point, concentrations were measured in the 0, 30, 100, 300 mg/kg groups. Sulfolane concentrations were increased in a greater than dose-dependent manner 2 h after the last dose administration and were similar between the two sexes. Similar trends were observed in both male and female rats, mice, and guinea pigs. At 2 h after the last dose, concentrations were generally consistent between rats and guinea pigs, while concentrations in mice were generally lower compared to the other two species. For example, at the 100 mg/kg dose, concentrations in rats and guinea pigs ranged from 109 to 115,000 ng/mL and 104–114,000 ng/mL respectively, while concentrations in mice ranged from 69 to 84,000 ng/mL. A small amount of sulfolane was detected in the plasma of rats and mice in the control group; however, this was likely due to background concentrations using this analytical method.

**Table 6:** Mean plasma concentrations (ng/mL) of sulfolane in male and female Sprague Dawley rats, B6C3F1/N mice, and Hartley guinea pigs at 2 h and 24 h after the last dose^a^.

Species/Sex	Hour	0 mg/kg	1 mg/kg	10 mg/kg	30 mg/kg	100 mg/kg	300 mg/kg	800 mg/kg
Male Rats	2	1.76 ± 1.13	---c	---	26033.3 ± 405.5	115333.3 ± 4630.8	543333.3 ± 63080.2	---
	24	1.32 ± 0.20	3.41 ± 1.42	8.56 ± 3.95	38.22 ± 8.54	8426.24 ± 2071.22	340300 ± 19619.2	720800 ± 36079.2
Female Rats	2	5.33 ± 1.80	---	–	31000.0 ± 2610.2	109000.0 ± 3214.6	461333.3 ± 32544.0	---
	24	2.38 ± 1.03	2.04 ± 0.35	5.70 ± 1.05	20.24 ± 3.72	3945.5 ± 1422.2	171800.0 ± 9756.8	594400.0 ± 55074.1
Male Mice	2	1.39 ± 0.77	---	----	4976.7 ± 841.6	83833.3 ± 3583.5	359333.3 ± 881.9	---
	24	BLOD^b^	BLOD	BLOD	BLOD	BLOD	7.99 ± 3.05	665142.9 ± 34001.7^d^
Female Mice	2	10.56 ± 1.20	–	–	7090.0 ± 1323.0	69266.7 ± 13182.1	354666.7 ± 7356.0	---
	24	BLOD	BLOD	BLOD	BLOD	2.32 ± 0.08	11.21 ± 3.89	739600.0 ± 17772.5^d^
Male Guinea Pigs	2	BLOD	---	---	25566.7 ± 584.1	114000.0 ± 2309.4	493333.3 ± 35890.3	---
	24	BLOD	BLOD	1.44 ± 0.25	6.47 ± 1.31	4662.7 ± 1625.7	214160.0 ± 63420.5	2290000.0 ± 780000.0^d^
Female Guinea Pigs	2	BLOD	---	---	24400 ± 321.5	104000.0 ± 1000.0	416666.7 ± 92851.7	---
	24	BLOD	BLOD	BLOD	6.37 ± 2.01	773.08 ± 305.6	150340.0 ± 30857.5	1214571.4 ± 98676.7^d^

a Data are displayed as mean ± SEM.

b Below the level of detection (BLOD) =1.25 ng/mL. Values below the LOD were substituted with ½ the LOD value if 20% or more of the values in a dose group were above the LOD. If 80% or more of the values in a dose group were below the LOD.

c Samples not collected in 1, 10, 800 mg/kg dose groups at the 2 h time point.

d Samples collected on day of moribund necropsy (male mice = 7; female mice = 5; male guinea pigs = 2; female guinea pigs = 7) and not 24 h after last dose.

For the 24 -h time point, plasma sulfolane concentrations were measured in core-study animals in all exposure groups (Table 6). Sulfolane was cleared rapidly 24 h after the last dose administration, and concentrations were very low or undetectable in the lower exposure groups (1 and 10 mg/kg) in all species. At 24 h in rats, concentrations in the 30 mg/kg groups decreased 681- and 1532-fold in males and females compared to the 2 -h time point. However, this difference decreased in magnitude at higher exposures with a 14- and 28-fold decrease in males and females respectively at 100 mg/kg and 2- to 3-fold decrease at 300 mg/kg. In mice, sulfolane concentrations were below the limit of detection at 24 h in the 30 and 100 mg/kg groups. In 300 mg/kg mice, sulfolane concentrations were decreased 45,000- and 32,000-fold in both males and females respectively compared to the 2 -h time point. Sulfolane concentrations in male and female guinea pigs were decreased 3800- and 4000-fold respectively in the 30 mg/kg group at 24 h relative to 2 h. Similar to the rats, the differences between 2 and 24 h decreased with dose (e.g., 24-fold and 2-fold difference at 100 and 300 mg/kg in male guinea pigs). In general, mice had lower concentrations at 24 h compared to rats and guinea pigs.

### 3.14 Micronuclei determinations

No significant increases in MN-RET or MN-E were observed in rats or mice of either sex. All values were within the laboratory historical control range. In mice, no significant alterations in the percentage of circulating RET among total erythrocytes were seen, suggesting that sulfolane did not induce bone marrow toxicity in mice. In rats, small increases in the percentage of circulating RET were noted, but these were well within the historical control 95% confidence interval and were of insufficient magnitude to be considered biologically significant. Summary data based on mean value per treatment group are provided in the supplementary data.

## 4 Discussion

Sulfolane is a high production volume (HPV) chemical used primarily as a solvent in extraction processes during refining [1,3,5,6]. Sulfolane has been detected in groundwater sources near refining sites, and the groundwater in North Pole, Alaska is known to be contaminated from a nearby petroleum refinery. Sulfolane concentrations of 4–7 parts per billion (ppb) have been detected in older supply wells [10] and groundwater monitoring has indicated concentrations up to 500 ppb [44]. Human health effects following sulfolane exposure are not well-characterized, and there are numerous challenges in conducting epidemiological studies for exposed communities, due in part to unknown information about past exposure or co-exposure [45]. EPA’s Provisional Peer-Reviewed Toxicity Values (PPRTV) for subchronic sulfolane exposure based on decreased white blood cell (WBC) counts in rats were 10 μg/kg/day and 1 μg/kg/day for chronic exposure [12]. To better ascertain species differences and similarities, NTP conducted 28-day toxicity studies to characterize the potential species and sex differences in sulfolane toxicity and aid in the interpretation of previous and future toxicity data.

Sulfolane exposure did not affect survival in rats in this study; however, mortality occurred in mice and guinea pigs administered 800 mg/kg sulfolane. Due to the high mortality rates, all mice and guinea pigs in the 800 mg/kg dose groups were euthanized early. The high dose in this study is roughly one-third to one-half of the reported LD_50_ values for acute sulfolane exposure for rats (1846 to 2342 mg/kg), mice (1900 to 2504 mg/kg), and guinea pigs (1445 to 1815 mg/kg) [1,2,15]). Interestingly, group mean body weights of 800 mg/kg male and female guinea pigs were decreased 9–14% while group mean body weights of 800 mg/kg male and female mice were increased 8–9% prior to early euthanasia. The group mean body weights of rats were decreased in 800 mg/kg males (∼10%) and 300 and 800 mg/kg females (∼7 and 8%, respectively).

Plasma sulfolane concentrations were assessed to determine species- and/or sex-specific differences in exposure 2 and 24 h after the last dose. Sulfolane concentrations generally increased with the dose at both time points in all sexes and species and were considerably lower at 24 h compared to 2 h. Taken together, these data are consistent with previously reported data that sulfolane is cleared rapidly [2,18]. Rats and guinea pigs had comparable plasma concentrations at both time points, while mice generally had lower concentrations compared to the other species at the same time point and dose, which is consistent with kinetic data [18,19]. While male and female rats had consistent concentrations at 2 h post-dosing, females generally had lower concentrations compared to males at 24 h, suggesting that sulfolane may be cleared somewhat/slightly faster in female rats after multiple dosing as this was not observed with single dose exposures [18]. Plasma concentrations between males and females within the mice and guinea pigs were consistent. There was evidence of nonlinear kinetics at the 2 -h and 24 -h time points as plasma concentrations in rats and guinea pigs were considerably higher in the 100 and 300 mg/kg groups on a dose-adjusted basis (ng/mL per mg/kg/day dose). It’s also noteworthy that the plasma concentrations in the mice and the guinea pigs in the 800 mg/kg group, which were removed early, were considerably high on a dose-adjusted basis (especially for the mice) indicating a saturation of metabolism/clearance pathways.

Sulfolane effects on WBC counts, the spleen, and other systems were of interest for this comparative study. A reported study assessing subchronic exposure to sulfolane in rats via drinking water observed decreases in WBC counts [26], while spleen effects were observed in guinea pigs in a different study [15]. In our study, a negative trend in WBC counts driven by decreases in lymphocyte counts was observed in both male and female rats; however, no WBC counts were statistically significant by pairwise comparison. This is somewhat consistent with the observations in female rats [26], but the magnitude of the effect was less, and it was unclear if the effect in the current study was related to a general stress response. There were no consistent effects on WBC counts in mice, while in guinea pigs lymphocytes counts were only statistically lower in the lowest dose groups (1 and 10 mg/kg/day groups). These leukocyte count changes in the mice and guinea pigs most likely represented biological variability. The lack of a dose response in guinea pigs along with plasma concentrations that were low or below quantification at 24 h suggests that this is not due to chemical effects. We did not observe an effect on spleen morphology in guinea pigs, as were observed in the [15]) study, but decreased spleen weights were observed in male and female rats. A downward trend in the erythrocyte count of rats and mice suggests a minimal effect on erythropoiesis in the higher dosed groups. The increase in the reticulocyte counts and the MCV (as reticulocytes are larger than erythrocytes) indicates an appropriate bone marrow response to decreases in red blood cells. No changes in bone marrow histology were observed and the reason for the mild changes in the erythron in this study is not known.

Absolute and/or relative liver weights were significantly increased in 300 and 800 mg/kg male and female rats. ALT activity, a biomarker for hepatocellular injury, was increased in 800 mg/kg male and female rats. These increases were mild, similar biomarkers (i.e., SDH) were unaffected, and no histologic hepatocellular lesions were observed, suggesting that these increases may be due to increased production (e.g., induction) by the liver. Triglycerides were increased in 800 mg/kg male rats, the mechanism of which is not known, and the significance of this finding is uncertain in light of the fact that it was not seen in the females or the guinea pig. There were no significant alterations in organ weights or clinical chemistry parameters in guinea pigs. Subchronic (6-month) studies in guinea pigs have reported a LOAEL of 2.5 mg/kg/day for oral sulfolane exposure that was partly based on changes in serum alkaline phosphatase (ALP) [15]; however, similar effects were not observed in our studies, which could be due to length of exposure or other design differences. It is not clear why the guinea pig was found to be more sensitive than the rat by [15] and not in the present study. Although the information reported was limited, the lack of similar effects between this study and the [15] study could be due to differences in design, including length of exposure and animal stock used.

In the male rats, there was a significant increase in hyaline droplet accumulation within the proximal tubule at all doses. This was also observed in a 90-day drinking water study in rats [19], along with tubular casts, which were not observed in this study. In addition to the hyaline droplet accumulation, kidney weights were increased in both male (300 and 800 mg/kg) and female rats (800 mg/kg) in this study suggesting a potential effect in females at high doses. Since the hyaline droplet accumulation was not seen in the female rats, or the other two species, this likely represents alpha 2U-globulin nephropathy. This is a male rat-specific change that may result in a low number of renal neoplasms in a chronic study. It is not clear, however, whether this mechanism is relevant to humans [48].

There were indications of gastrointestinal effects of sulfolane at the higher doses. In male mice, increases in chief cell hyperplasia and mineralization were observed in the glandular stomach, and hyperkeratosis was observed in the forestomach. In the glandular stomach of female mice, there was mineralization in the 800 mg/kg dose group that was similar to that seen in the male mice. Inflammation was also observed in the esophagus of male and female guinea pigs. There was also a low incidence of inflammation in the nasal cavity of male guinea pigs. The mechanism for these effects is unknown but may be due to direct contact and irritation of the gastrointestinal tract associated with the administration of the higher doses of sulfolane.

Sulfolane has been reported to induce central nervous system (CNS) toxicity in rats and mice at higher doses, with observed effects including hunched posture, hyperactivity, rapid breathing, and convulsions [2]. In our studies here, male and female rats in the 800 mg/kg group were observed with ruffled coats, hunched posture, and were cold to the touch; no signs of CNS toxicity were noted with the exception of a single 800 mg/kg female that showed seizure activity. There were no clinical signs of toxicity associated with the observed mortality in mice, while guinea pigs in the 800 mg/kg group exhibited lethargy, seizures, and hyperactivity prior to early euthanasia. Seizures were noted in 40% (4/10) of male guinea pigs administered 800 mg/kg sulfolane, which is higher than the reported NOAEL of 200 mg/kg and LOAEL of 400 mg/kg for seizure susceptibility in rats [46].

The reported neurotoxicity of sulfolane has been correlated with alterations in thermoregulation in rats and induction of regulated hypothermia in mice [2,24]. The thermoregulatory effects of sulfolane exposure may be due to the primary metabolite of sulfolane, 3-hydroxysulfolane, which has been proposed to act on the regions of the CNS that control thermoregulation [17]. In order to evaluate the effects of sulfolane exposure on thermoregulation, rectal temperatures were taken from all animals at study termination (or removed from study, i.e., 800 mg/kg mice and guinea pigs). Rectal temperatures of sulfolane-treated male or female rats were lower in the 800 mg/kg male and female groups, outside a reported normal range of 96.6–99.5 °F for rats (no strain/sex provided) [52]. Rectal temperatures collected from seven male and five female mice in the 800 mg/kg group prior to early euthanasia had lower temperatures and were outside of the normal range reported for mice (97.4–100.4 °F; no strain provided) [52], but a direct statistical comparison could not be conducted since controls and the 800 mg/kg group were removed at different times. Despite the significant mortality in 800 mg/kg guinea pigs, all mean rectal temperatures were similar to control values and within the normal range (99.0–103.1 °F; no strain provided) [52]. These data suggest species-specific differences in thermoregulatory effects following oral sulfolane exposure. Micronuclei (MN) have been reported to be elevated by high body temperature; however, no significant changes in MN-RET or MN-E were noted in these 28-day studies.

## 5 Conclusions

In this comparative study, guinea pigs appear to be the least sensitive species tested. They had similar plasma concentrations compared to rats at the 2- and 24 -h time points except for the 800 mg/kg group, where mortality occurred. A NOEL of 300 mg/kg for male and female guinea pigs was determined based on this lower survival. However, a NOEL of 30 mg/kg was determined for male mice based on observed mild decreases in erythrocyte counts and low incidences of chief cell hyperplasia and mineralization of the glandular stomach. Female mice had a NOEL of 100 mg/kg based on mild relative liver weight changes. Male rats appear to be the most affected with hyaline droplet accumulation occurring at all doses, although the human relevance of this finding is not clear. Aside from this kidney finding, the male rat NOEL was 100 mg/kg based on kidney and liver weight changes. For female rats, the NOEL was 10 mg/kg based on body weight decreases that occurred during the dosing period. These data indicate that the rat is the most sensitive of the three species tested.

Author Statement

All persons who meet authorship criteria are listed as authors, and all authors certify that they have participated sufficiently in the work to take public responsibility for the content, including participation in the concept, design, analysis, writing, or revision of the manuscript. Furthermore, each author certifies that this material or similar material has not been and will not be submitted to or published in any other publication before its appearance in *Toxicology Reports*.

## Declaration of Competing Interest

The authors report no declarations of interest.
